# Zoliflodacin inhibits *Streptococcus mutans* biofilms

**DOI:** 10.3389/fcimb.2025.1750551

**Published:** 2026-01-05

**Authors:** Muxin Xu, Jieyu Zhou, Linglong Lan, Haiyun Dong, Yunhe Shao, Gaozhe Zheng, Yangyang Pan, Yihuai Pan, Yan Sun

**Affiliations:** School and Hospital of Stomatology, Wenzhou Medical University, Wenzhou, China

**Keywords:** biofilm, cariogenic virulence, drug resistance, *Streptococcus mutans*, zoliflodacin

## Abstract

**Background:**

Zoliflodacin (ZFD) is a new type of antibiotic whose anticaries potential through controlling biofilms remains unknown. This study aimed to explore the effects of ZFD on the conditional formation of cariogenic *Streptococcus mutans* (*S. mutans*) biofilms.

**Methods:**

Crystal violet analysis, colony forming unit (CFU) counting, and MTT assays were used to determine biofilm biomass, viable bacterial cell counts, and bacterial metabolic viability, respectively, while acid generation by biofilms was monitored by measuring pH and lactic acid production. The anthrone-sulfuric acid method and polysaccharide/bacterial staining were used to investigate extracellular polysaccharide (EPS) production. The microstructure of *S. mutans* biofilms was observed using scanning electron microscopy (SEM). Growth curves were used to visualize bacterial growth, while live/dead bacterial staining was used to determine microbial activity. Furthermore, the cytotoxicity of ZFD was evaluated by live/dead cellular staining together with the CCK-8 assay, while drug resistance was induced using bacterial reduplicative exposure to ZFD. Quantitative real-time polymerase chain reaction (qRT-PCR) was used to calculate gene expression levels. In addition, checkerboard microdilution assays were performed to evaluate potential synergistic or antagonistic interactions of ZFD in combination with NaF or chlorhexidine (CHX) against bacterial growth and biofilm formation.

**Results:**

We found ZFD significantly inhibited *S. mutans* biofilm formation, decreasing overall biofilm biomass, viable bacterial cell counts within biofilms, and biofilm metabolic viability. ZFD not only markedly suppressed *S. mutans* cariogenic virulence involving acid and EPS synthesis, but also affected bacterial morphology within biofilms. Furthermore, ZFD inhibited bacterial growth without significantly suppressing the survival rate of bacterial cells within biofilms. Moreover, ZFD demonstrated no significant cytotoxicity and did not trigger drug resistance in *S. mutans* after 20 passages. qRT-PCR analysis revealed significant differences in the expression of virulence-, quorum sensing-, and oxidative stress protection-associated genes. Indifferent effects were found when ZFD was combined with NaF or CHX on *S. mutans* growth and biofilm formation.

**Conclusions:**

In summary, ZFD is a potential antibacterial agent for caries control because of its antibiofilm effects, inconspicuous cytotoxicity, low risk of resistance induction and antibacterial activity against *S. mutans*.

## Introduction

Dental caries has emerged as a severe global oral health issue owing to its high prevalence and notable treatment failure rates (Cheng et al., 2022). Between 1990 and 2021, the global number of untreated permanent dental caries increased by 53.0%, with a prevalence of 27.5% in 2021 (Eduardo et al., 2025). Clinically, dental caries manifests as chronic, gradual hard tissue destruction of the teeth. Considering that the disease is primarily biofilm dependent, prevention and treatment strategies should predominantly focus on targeting such biofilms (Liu et al., 2020). Among the numerous microorganisms within oral biofilms, *Streptococcus mutans* (*S. mutans*) is considered a primary pathogen of dental caries, mainly due to its array of virulence factors, including adherence capacity, extracellular polysaccharide (EPS) production, acidogenicity, and aciduricity, which substantially contribute to the formation and cariogenicity of dental biofilms (Krzyściak et al., 2014; Qiu et al., 2025). *S. mutans* facilitates initial adhesion and extracellular matrix accumulation, which are key steps in biofilm formation, by secreting glucosyltransferases (Gtfs) that synthesize EPS (Zhang et al., 2022b; Sun et al., 2024; Hayacibara et al., 2004). Concurrently, *S. mutans* produces large amounts of acidic metabolites via glycolysis, and EPS contribute to the maintenance of a localized acidic microenvironment. This acidic microenvironment triggers the acid tolerance response in *S. mutans*, thereby conferring a survival advantage to the bacterium (Matsui and Cvitkovitch, 2010; Guan and Liu, 2020). Consequently, this process exacerbates caries progression through continuous acid production (Luo et al., 2023). Moreover, bacteria residing within biofilm communities exhibit significantly enhanced resistance toward antibiotics (10 to 1000 times higher) than their planktonic counterparts owing to the defendable nature of the biofilm matrix (Haney et al., 2018; Costerton et al., 1999; Arciola et al., 2018). Therefore, *S. mutans* dental biofilms not only provide a protective niche for the bacteria against environmental stresses, but also sustain a localized acidic microenvironment conducive to tooth decay. Consequently, efficient strategies to control *S. mutans* biofilms are critical for caries control.

Despite the use of mechanical removal methods, various antimicrobial agents have been employed to manage biofilms and control the progression of caries. Among these, chlorhexidine (CHX) is widely regarded as the gold standard oral antiseptic owing to its broad-spectrum antibacterial properties and significant plaque-suppression capacity (Brookes et al., 2020). Nonetheless, its cytotoxicity and the potential to cause oral pain and numbness, taste alteration, tooth staining, tongue discoloration, xerostomia, dental calculus, and even a low incidence of hypersensitivity reactions remain concerns (Poppolo Deus and Ouanounou, 2022; Karpiński and Szkaradkiewicz, 2015). Another typical example is fluorides, which not only prevent caries through the remineralization of demineralized enamel but also inhibit the growth and acid production of cariogenic bacteria such as *S. mutans*. However, excessive fluoride exposure can lead to enamel hypomineralization, presenting as white chalky spots in mild cases and brown discoloration in severe instances (Nicholas et al., 2023). Notably, both CHX-resistant and fluoride-resistant oral microorganisms have been discovered, including *S. mutans* (Sun et al., 2024; Cieplik et al., 2019; Zhang et al., 2021). Drug resistance is a global challenge, and managing antimicrobial resistance-associated infections consumes 412 billion USD annually (Eduardo et al., 2025; Prestinaci et al., 2015). Consequently, it is necessary to seek novel antimicrobial agents that can efficiently suppress biofilm formation, including that of *S. mutans*, with few adverse effects and a low risk of resistance development.

Zoliflodacin (ZFD), a novel oral spiropyrimidinetrione-class antibiotic with activity against *Neisseria gonorrhoeae* (N. gonorrhoeae), is being developed for the treatment of gonorrhea (Taylor et al., 2018). It exhibits unique antibacterial activity by targeting bacterial type II topoisomerases, specifically acting on the GyrB subunit of DNA gyrase (Oyardi et al., 2024; Azam et al., 2015). Notably, ZFD shows no cross-resistance to commonly used clinical antibiotics and has not been associated with genotoxicity, bone marrow toxicity, or other significant adverse effects observed in certain fluoroquinolones (Bradford et al., 2020). In addition to those against *N. gonorrhoeae*, ZDF displays broad-spectrum inhibitory effects not only against *Staphylococcus, Streptococcus, Pseudomonas, Escherichia*, *Klebsiella*, *Acinetobacter*, *Enterococcus*, *Haemophilus*, and *Moraxella catarrhalis* including clinical drug-resistant strains (Biedenbach et al., 2015), but also against *Ureaplasma* and *Mycoplasma* species (Waites et al., 2015; Miller et al., 2019). Moreover, it shows cytotoxic effects toward *Chlamydia pneumoniae* and *C. trachomatis* (Miller et al., 2019; Kohlhoff et al., 2014). Furthermore, antibiofilm formation activities of ZFD against *Acinetobacter baumannii*, *Pseudomonas aeruginosa*, and *Klebsiella pneumoniae*, all of which are gram-negative pathogens, was recently reported (Oyardi et al., 2024). However, to date, no studies have reported the inhibitory effects of ZFD on biofilm formation by *S. mutans*, the major oral gram-positive cariogenic bacteria. Therefore, this study aimed to determine the antibiofilm effects of ZFD on *S. mutans*.

## Materials and methods

### Microbial strains and culture conditions

The *S. mutans* UA159 strain utilized in the present study was acquired from the School of Stomatology, Wenzhou Medical University. *S. mutans* UA159 was cultured overnight from a single-colony inoculant in brain heart infusion (BHI) medium at 37 °C in a 5% CO_2_ incubator. BHI medium was used in minimum inhibitory concentration (MIC) and growth curve assays, and an additional 1% (w/v) sucrose was added for biofilm-related assays to facilitate biofilm formation. Crystal violet staining, MIC, growth curve assays and checkerboard microdilution assay were performed in 96-well plates, while all other biofilm-related experiments were conducted in 24-well plates containing round glass coverslips. For all assays, culture medium with 10^6^ colony-forming units (CFU)/mL of *S. mutans* and varying doses of ZFD (InvivoChem, USA) were added to the wells at the beginning of the experiment and cultured for 24 h. A ZFD stock solution was prepared at a concentration of 25.6 mg/mL in dimethyl sulfoxide (DMSO, MP Biomedicals, USA). DMSO (0.5%) was used in the DMSO control group, whereas drug and DMSO-free wells served as the blank control group and 0.2% CHX was used in the positive control group.

### Crystal violet assay

To determine biofilm biomass, a crystal violet staining assay was performed after biofilm formation (Zhang et al., 2025). Briefly, 24-h *S. mutans* biofilms were formed in a 96-well plate and then washed with phosphate-buffered saline (PBS). Methanol was used to fix the biofilms (15 min), and a crystal violet solution (0.1%) was used to stain the biofilms (20 min). After washing away the non-specifically bound crystal violet dye using PBS, a stereomicroscope (Nikon SMZ800, Nikon Corporation, Japan) was used to observe the biofilms. To quantify biofilm formation, 33% acetic acid was added to dissolve the crystal violet bound to the biofilms (30 min), and the optical density (OD) at 590 nm was measured using a microplate reader (SpectraMax M5, Molecular Devices, USA).

### CFU counting assay

Viable bacteria in 24-h biofilms of *S. mutans* were quantitatively assessed using the CFU counting assay (Sun et al., 2021). Briefly, after removing the planktonic cells, each biofilm was mechanically scraped and collected in PBS. The suspension was vortexed to ensure uniform dispersion, and serially diluted in PBS. The diluted samples were plated onto BHI agar plates and incubated for 48 h at 37°C in a 5% CO_2_ environment. The viable bacterial count in each sample was determined by counting the number of colonies formed on the plates.

### MTT assay

The MTT assay was used to assess the effect of ZFD on *S. mutans* biofilm metabolic activity, as previously described (Zhang et al., 2022b). Briefly, 24-h biofilms in 24-well plates were mixed with 1 mL MTT solution (0.5 mg/mL), after washing with PBS, and then incubated at 37°C, protected from light, for 1 h. After incubation, the round glass coverslips with biofilms were transferred into new wells containing 1 mL DMSO. The plates were then incubated in the dark for 20 min, and 200 μL of the solution from each well was extracted before measuring the OD at 540 nm using a microplate reader (SpectraMax M5).

### pH measurement and lactic acid assay

After biofilm formation, the supernatants of the 24-h biofilms formed in 24-well plates were collected for pH measurement using a pH meter (Mettler Toledo Instruments Co., Ltd., China). For determining lactic acid production, the 24-h *S. mutans* biofilms were gently washed using cysteine peptone water and subsequently incubated with 1.5 mL of buffered peptone water containing 0.2% sucrose for 3 h at 37°C in a 5% CO_2_ atmosphere to maintain biofilm stability and promote lactic acid production. After 3 h, lactic acid concentration in the supernatant after acid production was assessed using an enzymatic method based on lactate dehydrogenase (Hu et al., 2022).

### Bacterial/EPS staining

To monitor EPS production and distribution within biofilms, bacterial/EPS staining in the biofilms was examined by confocal laser scanning microscope (CLSM). EPS in the biofilms were labeled with 2.5 μM Alexa Fluor 647-dextran (Invitrogen, USA), which was mixed with bacteria at the beginning of biofilm cultivation. After 24 h of biofilm development, the bacterial cells were labeled with 2.5 μM SYTO 9 (Invitrogen, USA) for 30 min. All stained biofilms were imaged using a confocal CLSM (Nikon A1, Nikon Corporation, Japan) with a 20× objective. The excitation wavelengths used for SYTO 9 and Alexa Fluor 647 were 480 and 650 nm, respectively, and their emissions were measured at 500 and 668 nm, respectively. Image-Pro Plus 6.0 software (Media Cybernetics, Inc., USA) was used to analyze the bacterial and EPS coverage areas in each biofilm layer (Zhang et al., 2022b).

### Water-insoluble glucan measurement

Water-insoluble EPS in the 24-h biofilms of *S. mutans* were quantified using the anthrone method (Sun et al., 2019). After removing planktonic cells, biofilms in each well were resuspended in 0.4 M sodium hydroxide and incubated at 37°C for 30 min. Following centrifugation, biofilm supernatants (100 μL each) and anthrone reagent (300 μL) were mixed and incubated for 6 min at 95°C. The OD was measured at 625 nm using a microplate reader (SpectraMax M5). WIG content was determined based on a standard curve.

### Scanning electron microscopy imaging

Morphological changes in *S. mutans* under different treatment conditions were examined under SEM (Zheng et al., 2013). After 24-h biofilm formation, the samples were fixed with glutaraldehyde (2.5%) for 8 h and then dehydrated using a series of ethanol solutions (50%, 60%, 70%, 80%, 90%, 95%, and 100%); each concentration was applied for 30 min. After dehydration, the samples were dried using the critical point drying method and gold-coated for surface examination. The morphological characteristics of the biofilms were imaged using a SEM (Hitachi, Japan) at magnifications of 2,000× and 10,000×.

### Live/dead bacterial viability assay

Briefly, SYTO 9 (2.5 μM, green dye for live bacteria) and propidium iodide (PI) (2.5 μM, red dye for dead bacteria) were used to stain live and dead bacterial cells, respectively, within 24-h biofilms for 30 min. Fluorescence images were captured using a confocal CLSM (Nikon A1) in random fields with a 20× objective. The excitation and emission wavelengths for SYTO 9 were 480 nm and 500 nm, respectively, while those for Alexa Fluor 647 were 490 nm and 635 nm, respectively. The percentage of live bacteria in each biofilm was calculated using Image-Pro Plus 6.0 software (Media Cybernetics, Inc., USA) based on the coverage of five randomly selected visual fields in each group (Zhang et al., 2022b).

### Growth curve determination assay

Growth curve determination assays were used to monitor bacterial growth. Overnight-cultured *S. mutans*, at a concentration of 10^6^ CFU/mL, was mixed with various concentrations of ZFD, 0.5% DMSO, or 0.2% CHX, and cultured for 24 h at 37°C. The OD of the cultures at 600 nm was measured at 1.5-h intervals using a microplate reader to generate the bacterial growth curve (Zhang et al., 2022b).

### Cytotoxicity assays

To monitor cytotoxicity of ZFD, cytotoxicity assays were conducted mainly including cell live/dead imaging and cellular viability determination (Hu et al., 2022). For cell live/dead imaging, L929 mouse fibroblasts (10^4^ cells/well) were mixed with 100 µL DMEM supplemented with 10% fetal bovine serum and cultured for 24-h at 37°C and 5% CO_2_ to allow attachment to the wells of 96-well plates. After incubation, the medium was replaced using 100 μL of different concentrations of ZFD (0, 0.250, 0.125, 0.063, or 0.031 μg/mL), 0.5% DMSO, or 0.2% CHX and incubated for an additional 24 h period at 37°C and 5% CO_2_. The cells were then stained with 100 μL of Calcein-AM (green dye for live cells) and 100 μL of PI (red dye for dead cells) at 37°C for 30 minutes. Fluorescent images were captured using an inverted fluorescence microscope (Axio Vert.A1, Zeiss, Germany).

Cell viability was evaluated using the cell counting kit-8 (CCK-8) assay (Dojindo, Japan). After treating cells under the same conditions than those for attachment assays and with the same drug treatment than that used in cell live/dead imaging, CCK-8 reagent (10 μL) was incorporated to each well and incubated at 37°C for 1 h. A microplate reader (SpectraMax M5) was used to measure the OD at 450 nm.

### Bacterial drug resistance assay

To evaluate whether *S. mutans* can develop resistance after prolonged exposure to antimicrobial agents, the MIC was determined, followed by the repeated sequential passage method (Zhang et al., 2021). The MIC was determined using the microdilution method (Zhang et al., 2021). Briefly, serial dilutions of the test drugs, including ZFD and CHX, were mixed with overnight *S. mutans* culture aliquots (final concentration 10^6^ CFU/mL) and incubated at 37 °C and 5% CO_2_ for 24 h. The MIC was defined as the lowest antibacterial concentration at which no visible bacterial growth or turbidity was observed in 200 μL culture. Subsequently, 100 μL culture suspensions in sub-MIC (0.5 MIC) concentrations were transferred into fresh medium (10 mL) and incubated overnight. The following day, the culture was diluted to a final concentration of 10^6^ CFU/mL and used for subsequent rounds of MIC determination. This process was repeated 20 times to monitor the development of drug resistance.

### RNA isolation and quantitative real-time polymerase chain reaction

Total RNA was isolated from 24-h biofilms using TRIzol reagent (Invitrogen, USA) as previously described (Zhang et al., 2025). Afterwards, RNA was reverse transcribed into cDNA using a PrimeScript™ RT master mix kit (Takara, Japan). qRT-PCR was performed using a TB Green™ Premix Ex Taq™ II (Tli RNaseH Plus) kit (Takara) on a Step One Plus real-time PCR system (Applied Biosystems, USA) as previously described (Sun et al., 2021). The 16S rRNA gene was used as the reference gene (the primers used are listed in **Table 1**). Relative gene expression was determined using the 2^-ΔΔCt^ method. The DMSO group was used as the control group.

**Table 1:** Primers used in this study.

Primers	Nucleotide sequence (5'-3')	References
*16S-*f	CCTACGGGAGGCAGCAGTAG	(Zhang et al., 2022b)
*16S-*r	CAACAGAGCTTTACGATCCGAAA	
*gtfB-*f	AGCAATGCAGCCAATCTACAAAT	(Zhang et al., 2022b)
*gtfB-*r	ACGAACTTTGCCGTTATTGTCA	
*gtfC-*f	CTCAACCAACCGCCACTGTT	(Zhang et al., 2022b)
*gtfC-*r	GGTTTAACGTCAAAATTAGCTGTATTAGC	
*gtfD-*f	ACAGCAGACAGCAGCCAAGA	(Zhang et al., 2022b)
*gtfD-*r	ACTGGGTTTGCTGCGTTTG	
*ldh-*f	AAAAACCAGGCGAAACTCGC	(Zhang et al., 2022b)
*ldh-*r	CTGAACGCGCATCAACATCA	
*nox-*f	GGGTTGTGGAATGGCACTTTGG	(Feldman et al., 2016)
*nox-*r	CAATGGCTGTCACTGGCGATTC	
*sodA-*f	GCAGTGCTAAGACTCCCGAATC	(Feldman et al., 2016)
*sodA-*r	TTGCGGAAGTGTGAGATTGGC	
*comX-*f	CGTCAGCAAGAAAGTCAGAAAC	(Sun et al., 2021)
*comX-*r	ATACCGCCACTTGACAAACAG	
*luxS-*f	ACTGTTCCCCTTTTGGCTGTC	(Zhang et al., 2022b)
*luxS-*r	AACTTGCTTTGATGACTGTGGC	
*comDE-*f	ACAATTCCTTGAGTTCCATCCAAG	(Zhang et al., 2022b)
*comDE-*r	TGGTCTGCTGCCTGTTGC	

### Checkerboard microdilution assay

Checkerboard microdilution assays were performed to determine potential synergistic/antagonistic outcomes of the combination of ZFD with NaF or CHX on bacterial growth and biofilm formation (Zheng et al., 2015). To facilitate quantitative analysis, MIC_90_ and minimum biofilm inhibitory concentration_90_ (MBIC_90_) were used. MIC_90_ and MBIC_90_ were determined as the lowest drug concentration that resulted in at least 90% reduction in bacterial growth and biofilm formation respectively. Briefly, cultures containing 10^6^ CFU/mL of *S. mutans* and varying doses of drugs or drug combinations were incubated for 24 h. ZFD concentrations ranged from 0.250 to 0.016 μg/mL, NaF from 500 to 31.25 ppm, and CHX from 1.56 to 0.098 μg/mL. For bacterial growth detection, BHI medium was used and OD at 600 nm was detected using a microplate reader (SpectraMax M5). The fractional inhibitory concentration index (FICI) was determined as the sum of the ratios of the MIC_90_ of each drug in combination to its MIC_90_ alone (FICI = MIC_90_ of drug A (in combination)/MIC_90_ of drug A (alone) + MIC_90_ of drug B (in combination)/MIC_90_ of drug B (alone)). The final results were classified as a synergistic effect (FICI ≤0.5), an additive effect (0.5< FICI <1), an indifferent effect (1≤ FICI <4); and an antagonistic effect (FICI ≥4). For biofilm formation, potential synergistic/antagonistic outcomes of drug combinations against biofilm were evaluated similarly, except that additional 1% (w/v) sucrose was added to BHI medium, and the minimum biofilm inhibitory concentration (MBIC_90_) was determined instead of the MIC_90_.

### Statistical analysis

All experiments were independently repeated three times, and data were expressed as the mean ± standard deviation (SD). Statistical significance was assessed using one-way analysis of variance (ANOVA). Statistical significance was defined as follows: non-significant (NS) *P* ≥ 0.05 and significant * *P* < 0.05, ** *P* < 0.01, and *** *P* < 0.001.

## Results

### ZFD inhibits *S. mutans* biofilm formation

**Figure 1:**
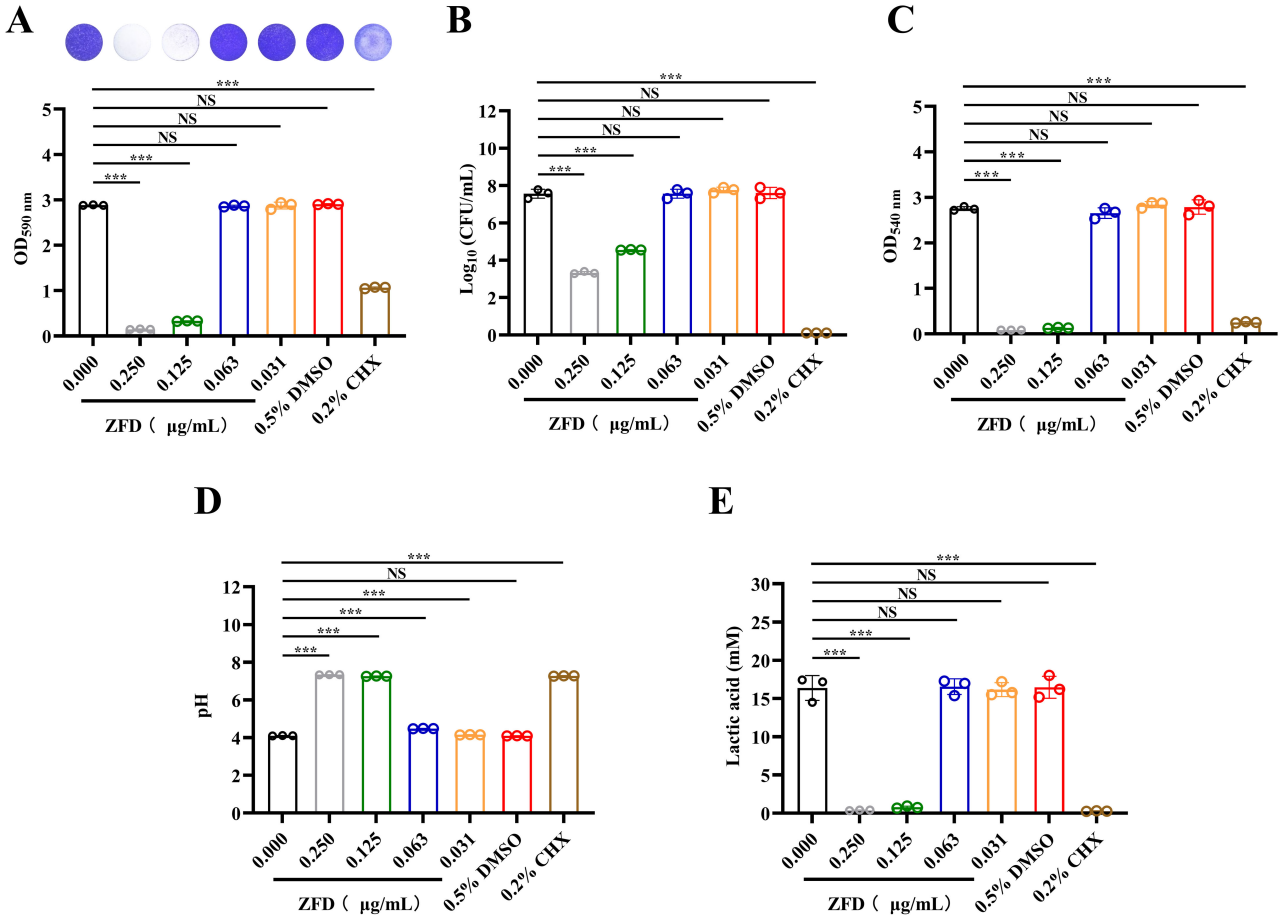
Biomass, CFU counts, metabolic viability, and acid production of *S. mutans* biofilms. **(A)** Relative biofilm biomass of *S. mutans* biofilms assessed by crystal violet staining; **(B)** CFU counts of viable *S. mutans* within biofilms; **(C)** Metabolic activity of *S. mutans* biofilms evaluated using the MTT assay; **(D)** Supernatants pH of biofilms; **(E)** Quantification of lactic acid production by *S. mutans* biofilms. (***P < 0.001; NS, non-significant).

ZFD (0.250 μg/mL and 0.125 μg/mL) showed a significant inhibitory effect on the formation of *S. mutans* biofilms, as determined by crystal violet staining (*P* < 0.001) (**Figure 1A**). Conversely, these significant inhibitory effects were not found at lower concentrations of ZFD (0.063 μg/mL and 0.031 μg/mL) (*P* > 0.05). Results from CFU counting and MTT assays corroborated the findings of the crystal violet staining assay (*P* < 0.001) (**Figures 1A–C**). The DMSO control group did not exhibit a significant effect on biofilm formation, whereas 0.2% CHX, which was used as a positive control, demonstrated a pronounced antibiofilm effect (*P* < 0.001). These results indicate that ZFD’s inhibitory effect on *S. mutans* biofilm formation is dose-dependent and more effective at higher concentrations.

### ZFD suppresses *S. mutans* biofilm acid production

pH measurement results revealed that ZFD effectively restricted acid production in *S. mutans* biofilms (**Figure 1D**). ZFD treatments, either with 0.250 μg/mL or 0.125 μg/mL, significantly elevated the supernatant pH to above 7 (*P* < 0.001), which was similar to that observed with 0.2% CHX. These ZFD concentrations also reduced lactic acid production by 97.6% and 95.2%, respectively, compared to that of the blank control group (*P* < 0.001). In contrast, at lower concentrations of ZFD (0.063 μg/mL and 0.031 μg/mL), the pH values of the ZFD treatment groups were only a little higher than that of the blank control group (*P* < 0.001), and they both remained at pH values below 5.5, which is the critical threshold for enamel demineralization. A similar trend was observed regarding lactic acid production, and the changes observed at the lower concentrations of ZFD were minimal and not statistically significant compared to the blank control values (*P* > 0.05). These results indicate that ZFD’s inhibitory effect on *S. mutans* biofilm lactic acid production is also dose-dependent, and the most pronounced effects were observed at higher ZFD concentrations.

### ZFD hinders EPS production in *S. mutans* biofilms

**Figure 2:**
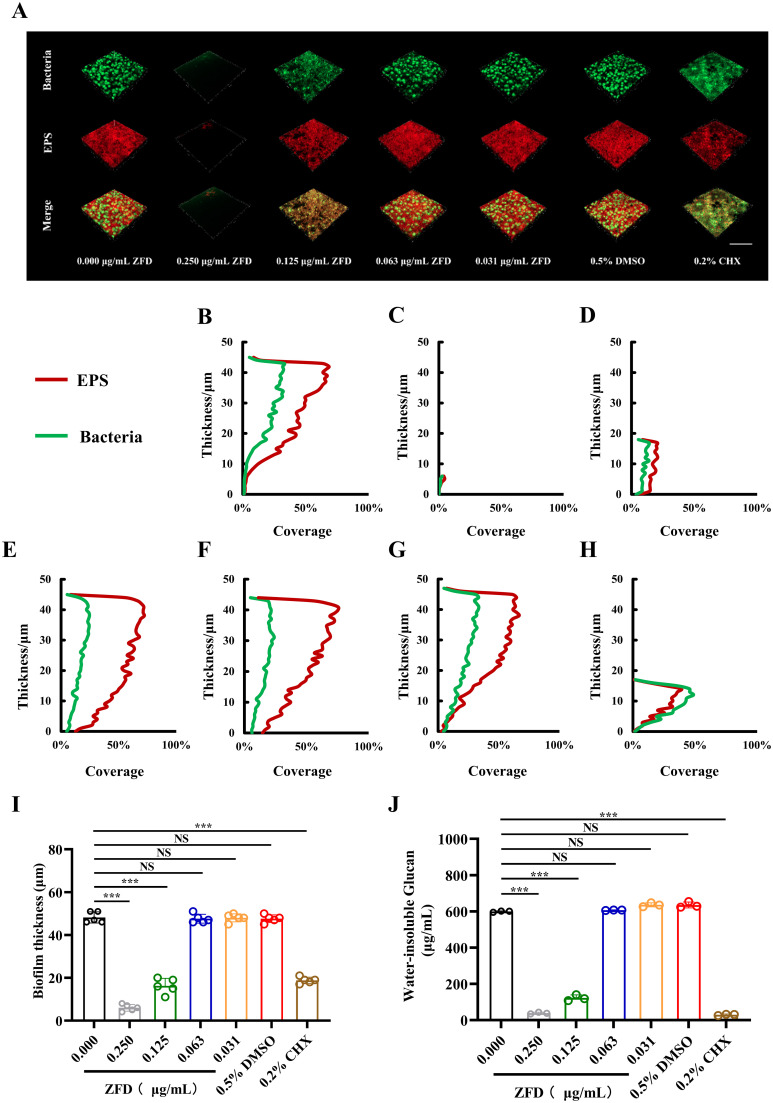
EPS staining, biofilm thickness and WIG synthesis of *S. mutans* biofilms. **(A)** CLSM images of EPS’ distribution in *S. mutans* biofilms under ZFD, Green fluorescence (SYTO 9) represents bacteria, and red fluorescence (Alexa Fluor 647) represents EPS, Bar = 100 μm; Bacterial and EPS area coverage in each biofilm layer under **(B)** 0.000 μg/mL ZFD, **(C)** 0.250 μg/mL ZFD, **(D)** 0.125 μg/mL ZFD, **(E)** 0.063 μg/mL ZFD, **(F)** 0.031 μg/mL ZFD, **(G)** 0.5% DMSO, and **(H)** 0.2% CHX; **(I)** Biofilm thickness of *S. mutans* biofilms; **(J)** Quantification of water-insoluble glucans in *S. mutans* biofilms. (***P < 0.001; NS, non-significant).

As shown in **Figure 2A**, the reconstructed three-dimensional biofilms displayed much less EPS under 0.250 μg/mL and 0.125 μg/mL ZFD which confirmed its inhibiting effect on *S. mutans* biofilm EPS production. Likewise, significant reduction in biofilm EPS was also found in CHX group. Subsequent layer-by-layer analysis revealed that treatment with ZFD at 0.250 μg/mL and 0.125 μg/mL significantly reduced both bacterial clusters and EPS within *S. mutans* biofilms, as evidenced by a decrease in the coverage of bacterial cells and EPS along the z-axis (**Figures 2B–H**). As a result, the thickness of the reconstructed three-dimensional *S. mutans* biofilms was decreased (**Figure 2I**) (*P* < 0.001). Additionally, anthrone assay results corroborated that the content of WIG in *S. mutans* biofilms treated with 0.250 μg/mL and 0.125 μg/mL ZFD was statistically lower than that in the blank control group (**Figure 2J**) (*P* < 0.001). No significant difference regarding WIG production was found between the DMSO and control groups (*P* > 0.05).

### ZFD affects bacterial morphology within *S. mutans* biofilms

**Figure 3:**
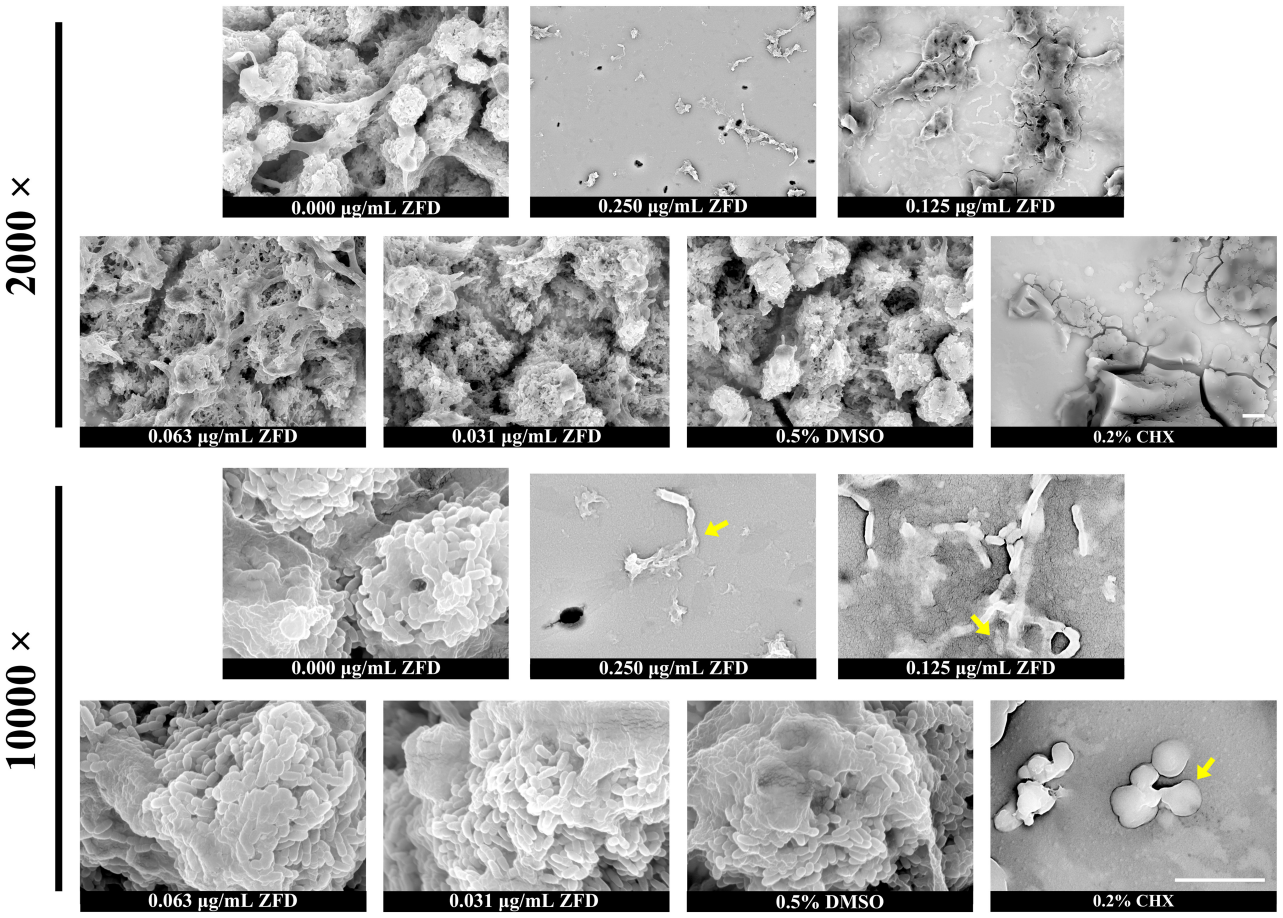
SEM images of *S. mutans* biofilms. Yellow arrows indicate representative abnormal bacterial cell. Images were captured at 2,000 × and 10,000 × magnifications. Bar = 5 μm.

Bacterial morphological analysis was performed by SEM imaging. After treatment with 0.250 μg/mL and 0.125 μg/mL ZFD, significant bacterial morphological changes were observed, along with a marked reduction in EPS accumulation (**Figure 3**). The extent of morphological distortion in cells from the 0.250 μg/mL group was more severe than that observed in cells of the 0.125 μg/mL group. In contrast, at lower concentrations of ZFD (0.063 μg/mL and 0.031 μg/mL), the structure of bacterial cells remained intact and neat edges without wrinkles could be observed. These results suggest that, at concentrations of 0.250 μg/mL and 0.125 μg/mL, ZFD alters the surface structure and morphology of *S. mutans* cells. In addition, *S. mutans* cells from the 0.2% CHX group displayed a swollen and fractured morphology.

### ZFD inhibits bacterial growth without significantly suppressing the survival rate of *S. mutans* within biofilms

**Figure 4:**
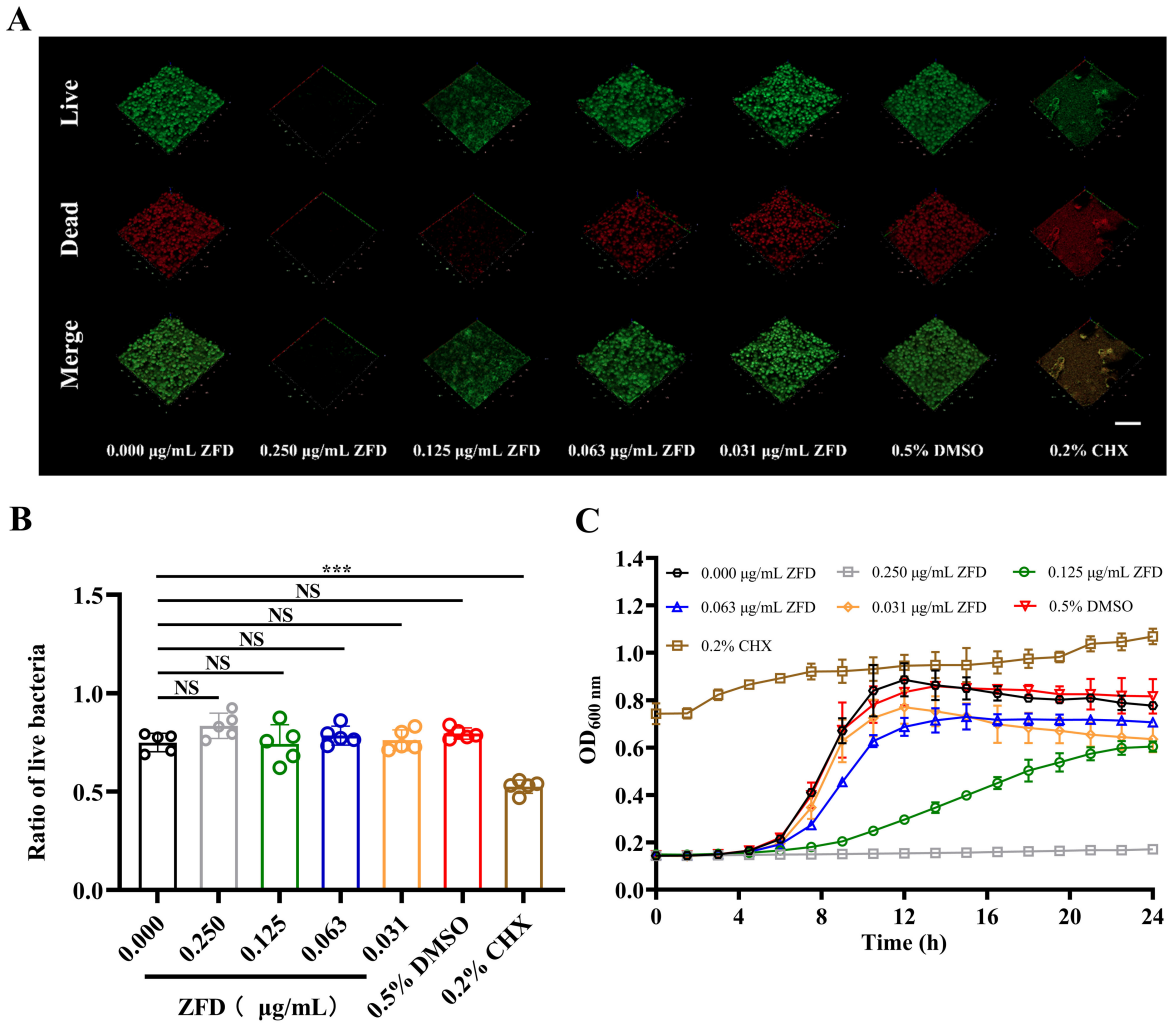
Live/dead staining of biofilms and bacterial growth curves. **(A)** CLSM images of live/dead *S. mutans* distribution within biofilms. Live and dead bacteria are shown in green (SYTO 9) and red (PI), respectively. Bar = 100 μm; **(B)** Proportion of live bacteria in the biofilm based on live/dead staining; **(C)** 24-hour growth curves of *S. mutans*. (***P < 0.001; NS, non-significant).

Following treatment with different concentrations of ZFD, bacterial viability was assessed by live/dead staining. Treatment with 0.125 μg/mL and 0.250 μg/mL ZFD resulted in a considerable reduction in the number of both live and dead bacteria compared to those in the other groups. Notably, at concentration of 0.250 μg/mL ZFD, almost no live or dead bacteria were detected, indicating a marked inhibition of biofilm formation (**Figure 4A**). However, statistical analysis of the live bacteria proportion in *S. mutans* biofilms showed no significant differences among the groups regardless of the ZFD concentration used (**Figure 4B**).

Growth curve results showed that, as the concentration of ZFD increased, *S. mutans* growth was significantly suppressed. At concentrations of 0.250 μg/mL ZFD, bacterial growth was almost completely inhibited, whereas, 0.125 μg/mL ZFD prolonged the lag phase and reduced maximum growth density (**Figure 4C**). However, there was no significant difference between the blank and DMSO controls. These findings further confirmed that the inhibitory effect of ZFD on *S. mutans* biofilm formation occurred, at least in part, through growth inhibition.

### ZFD exhibits inapparent cytotoxicity

**Figure 5:**
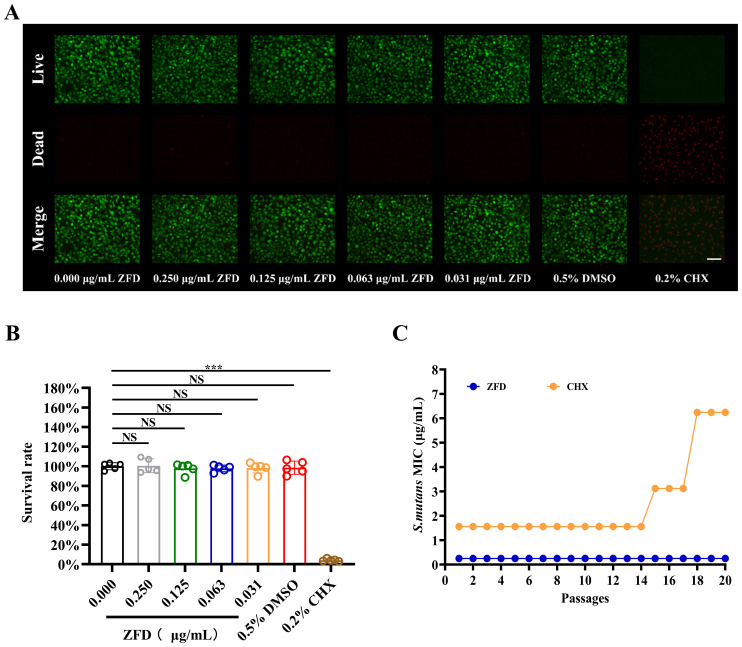
Cytotoxicity of ZFD and MICs of drugs against *S. mutans* with serial passage. **(A)** Representative inverted fluorescence microscopy images of L929 cells. Live cells are stained green with Calcein-AM, and dead cells are stained red with propidium iodide (PI). Bar = 100 μm; **(B)** Cellular survival rate determined by the CCK-8 assay; **(C)** MIC of ZFD and CHX against *S. mutans* over 20 consecutive passages. (***P < 0.001; NS, non-significant).

To evaluate the cytotoxicity of ZFD, cell viability was assessed using live/dead staining images. No remarkable difference between the ZFD-treated and control groups was observed in terms of viability, and most cells were viable. In contrast, the 0.2% CHX positive control group showed almost no viable cells, indicating superior cytotoxicity (**Figure 5A**). Additionally, cytotoxicity was evaluated using the CCK-8 assay on mouse fibroblasts (L929) at different ZFD concentrations. Similarly, no significant difference in cell viability was observed between the ZFD-treated and control groups (**Figure 5B**). In contrast, a significantly lower cell survival rate was observed in the positive control group (*P* < 0.001). Collectively, these results demonstrate that ZFD exhibits inapparent cytotoxicity.

### ZFD does not trigger drug resistance against *S. mutans*

At subinhibitory concentrations of ZFD, the MIC against *S. mutans* remained stable after 20 consecutive passages (**Figure 5C**). The MIC value for the ZFD-treated group consistently remained at 0.250 μg/mL throughout passages 0 to 20, showing no fluctuations. These results implied that frequent exposure to ZFD did not lead to increased resistance in *S. mutans*, demonstrating favorable stability against resistance development. In contrast, the CHX-treated group exhibited a marked increase in MIC after passage 15, indicating a clear trend toward resistance induction.

### ZFD impacts *S. mutans* gene expression within biofilms

**Figure 6:**
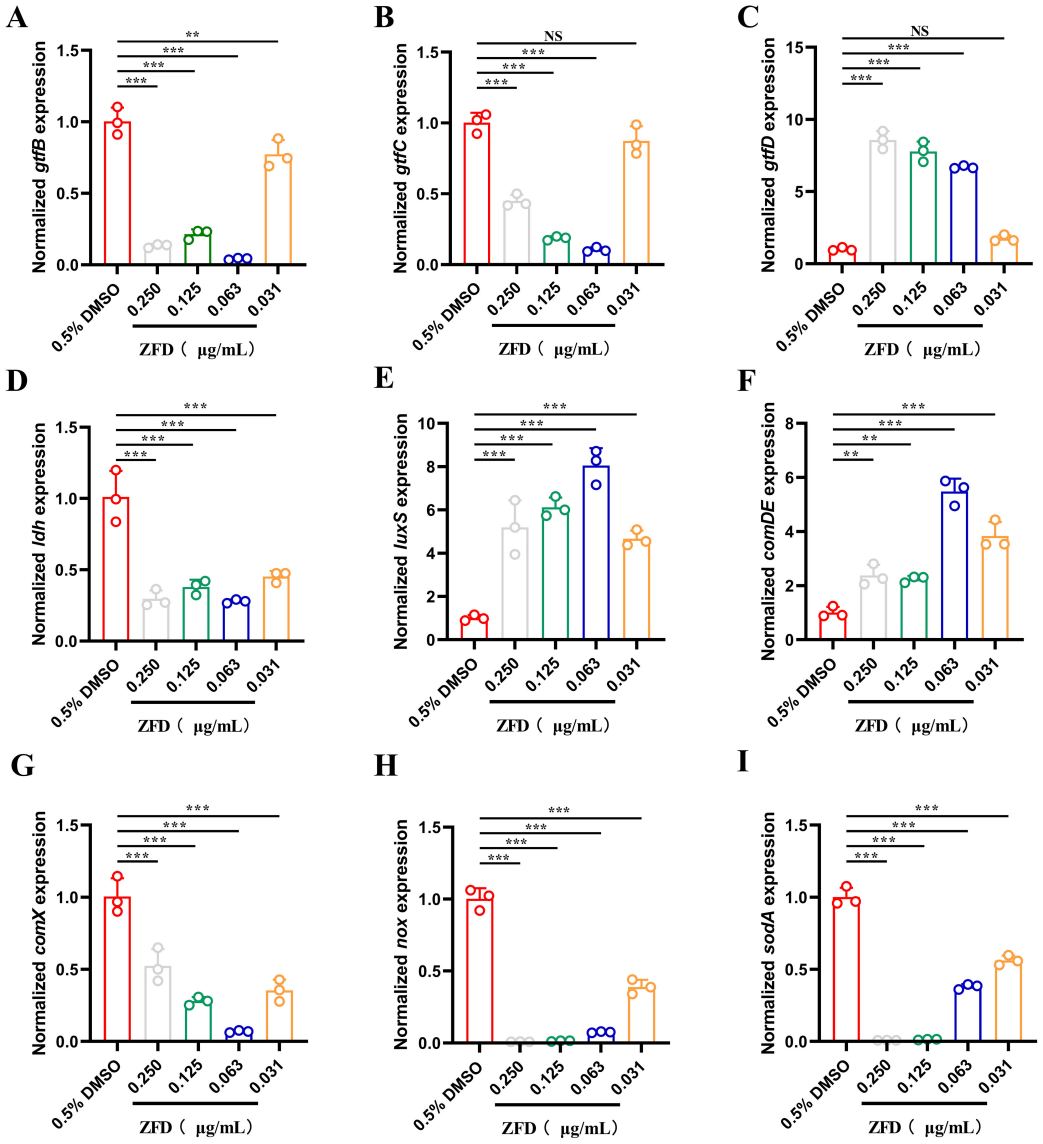
qRT-PCR analysis of *S. mutans* genes. Expression level of **(A)***gtfB*, **(B)***gtfC*, **(C)***gtfD*, **(D)***ldh*, **(E)***luxS*, **(F)***comDE*, **(G)***comX*, **(H)***nox*, **(I)***sodA* in *S. mutans* biofilms. (**P < 0.01; ***P < 0.001; NS, non-significant).

Within biofilms, the expression of polysaccharide synthesis-associated genes exhibited different trends. While the expression of *gtfB* and *gtfC* (**Figures 6A, B**) in *S. mutans* biofilms was significantly downregulated (except for that of *gtfC* under 0.031 μg/mL ZFD), that of *gtfD* (**Figure 6C**) was upregulated (except under 0.031 μg/mL ZFD) when compared with the expression observed in the DMSO control group. Furthermore, treatment with ZFD at all tested concentrations resulted in decreased expression of the *ldh* gene (**Figure 6D**), which was linked to lactic acid production. In addition, the expression of *luxS* (**Figure 6E**) and *comDE* (**Figure 6F**), which were related to bacterial quorum sensing (QS), was substantially upregulated in all the ZFD groups. In contrast, compared to that in the control groups, the expression of the *comX* gene (**Figure 6G**), which controlled the expression of late-competence genes, was significantly lower in ZFD-treated groups. Additionally, the expression of *nox* —a major contributor to the oxidative stress response, reducing ROS production— (**Figure 6H**) and *sodA* —the superoxide dismutase gene— (**Figure 6I**) was significantly downregulated in the ZFD-treated groups.

### Combinations of ZFD with NaF or CHX showed indifferent effects on *S. mutans* growth and biofilm formation

**Figure 7:**
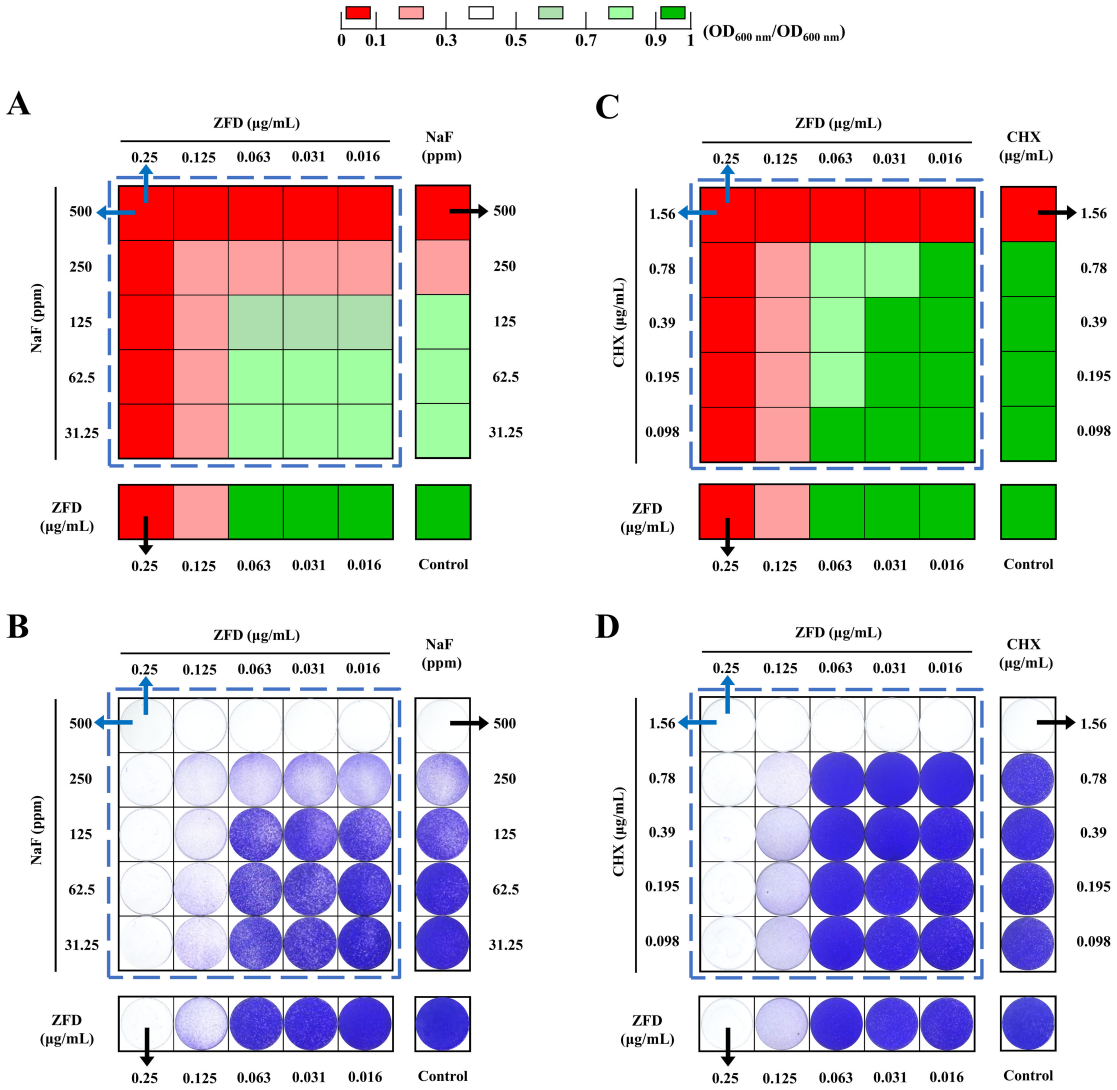
Combination of ZFD with NaF or CHX on growth and biofilm formation of *S. mutans*. **(A)** Combination of ZFD with NaF on growth of *S. mutans*, **(B)** combination of ZFD with NaF on *S. mutans* biofilms, **(C)** combination of ZFD with CHX on growth of *S. mutans*, **(D)** combination of ZFD with CHX on *S. mutans* biofilms. Results for drug combinations are shown inside the dotted boxes, whereas results for single drug are shown outside. Data were normalized to the corresponding DMSO controls. Blue arrows indicate MIC_90_s or MBIC_90_s for drug combinations, whereas black arrows indicate MIC_90_s or MBIC_90_s for single-agent treatments.

ZFD combined with NaF or CHX showed indifferent effects against *S. mutans* planktonic cultures, as the FICI was 2 for both combinations (**Figure 7**). Likewise, ZFD combined with NaF or CHX also showed indifferent effects on *S. mutans* biofilm formation, based on their FICI values (FICI=2, **Figure 7**).

## Discussion

In our study, we systematically reported the dose-dependent inhibitory effect of ZFD on biofilm formation and cariogenic virulence, involving acid and EPS production, of *S. muta*ns, a primary oral gram-positive cariogenic bacterium. ZFD affected bacterial morphology and inhibited biofilm formation by restricting bacterial growth but not bacterial activity. No significant cytotoxicity or drug resistance induction was observed among *S. mutans* ZFD-treated groups. Moreover, significant differences were found in the expression of genes associated with virulence, QS, and oxidative stress protection. In addition, indifferent effects on *S. mutans* growth and biofilm formation were found for combinations of ZFD with NaF or CHX. Previous studies have demonstrated that ZFD inhibits biofilm formation by gram-negative pathogens. Based on our findings, we conclude that ZFD can suppress biofilm formation in both gram-negative and gram-positive bacteria.

The acidogenic capability of *S. mutans* is one of its major virulence factors, which is associated with cariogenicity (Li et al., 2016). This organism persistently colonizes the oral environment and continuously produces organic acids through carbohydrate metabolism. Highly acidic environments can give a survival advantage to acid-producing and acid-tolerant bacteria such as *S. mutans*, resulting in an imbalance in dental plaque. Meanwhile, acidic environments also lead to hydroxyapatite dissolution and calcium and phosphate ion loss, disrupting the balance between demineralization and remineralization and ultimately initiating dental caries at pH < 5.5 (Marsh, 2003). Lactate dehydrogenase (LDH), which is encoded by *ldh*, is critical for glycolysis and acid production in *S. mutans* (Sun et al., 2024). Its product, lactic acid, accounts for approximately 70% of the total organic acids produced in oral biofilms (Melo et al., 2016). In this study, we observed a significantly lower *ldh* expression in the ZFD group than in the DMSO control group. Lactic acid’s quantitative determination further revealed that treatment with 0.25 μg/mL and 0.125 μg/mL ZFD significantly reduced lactic acid levels. These findings suggest that ZFD may inhibit the glycolytic acidogenic process by downregulating *ldh* expression and reducing lactic acid production, thereby preventing the establishment of an acidic microenvironment. Consistent with lactic acid level analysis results, the pH of the supernatant was significantly higher in the ZFD-treated groups than in the control groups. It should be noted, however, that the decrease in lactic acid concentrations and the increase in pH may also be partially attributed to the reduced number of viable bacterial cells, which may diminish the overall acidogenic potential of the cultures.

Within oral biofilm, *S. mutans* is mainly an EPS manufacturer. This EPS, mainly composed of WIG, is the primary structural component of the biofilm; it creates a complex polysaccharide network that enhances bacterial adhesion and provides localized acidic microenvironments, thereby promoting bacterial survival and metabolic activity and increasing biofilm pathogenicity (Sun et al., 2021; Chen et al., 2020). Therefore, EPS is regarded as an important cariogenic virulence factor (Klein et al., 2015). Our results showed that much less WIG was generated in 0.125 μg/mL and 0.250 μg/mL ZFD-treated cultures suggesting that ZFD may suppress biofilm formation by reducing WIG production and, consequently, impairing the adhesive capability of *S. mutans*. The reduction in WIG may partly result from the downregulation of *gtfB* and *gtfC*, which encode GtfB —responsible for synthesizing WIG, and GtfC —which synthesize both WIG and water-soluble glucans) (Kim et al., 2025; Zhao et al., 2014). Moreover, similar to lactic acid reduction results, WIG’s decrease may also partly result from the decrease in bacterial cell number within biofilms. In addition to GtfB and GtfC, another glucosyltransferase, GtfD (encoded by *gtfD*), primarily produces water-soluble glucans, which can serve as energy storage resources (Koo et al., 2013). Notably, *gtfD* expression was upregulated in cultures treated with ZFD, which may have resulted from increased energy requirements to combat antibiotic stress.

QS is an intercellular communication mechanism based on the generation, release, and monitoring of signaling molecules. It enables bacteria such as *S. mutans* to monitor population density and coordinate group behaviors, including biofilm formation, genetic competence, and virulence factor expression (Feldman et al., 2016). Autoinducer 2 (AI-2)/LuxS and ComCDE (which uses competence stimulating peptide as its signal molecule) are two well-identified QS systems that are responsible for inter- and intra-species communication, respectively (Smith and Spatafora, 2012). S-ribosylhomocysteine lyase (LuxS), which is encoded by *luxS*, has a dual function; it is responsible for AI-2 synthesis and *S*-adenosylhomocysteine detoxification (Sztajer et al., 2008). ComD, encoded by *comD*, is a histidine kinase, whereas ComE, encoded by *comE*, is a response regulator. It has been reported that both *luxS* and *comDE* are essential for robust biofilm formation and that any deficiency would result in biofilm formation abatement (Sztajer et al., 2008; Li et al., 2002). In this study, we observed that treating *S. mutans* with ZFD significantly upregulated the expression of QS-related genes, including that of *luxS* and *comDE*. This response may result from bacteria trying to upregulate biofilm formation-related genes to cope with the inhibitory effects of ZFD on biofilm formation (Sun et al., 2021). However, despite the upregulation of upstream pathways, the expression of *comX* was notably suppressed in the ZFD-treated groups. ComX is a key downstream regulator of *comCDE*, and its activation may induce bacterial competence and autolysis (Zu et al., 2019), which may be related to the cross-communication regulatory systems engaged. For example, the expression of *comX* is also controlled via other signaling systems such as the ComRS regulatory circuit. Importantly, *S. mutans* tends to reduce bacterial lysis to combat ZFD stress (Zu et al., 2019). Interestingly, decreased expression of oxidative stress protection-associated *nox* (NADH oxidase) and *sodA* (superoxide dismutase) was observed. The downregulation of these genes suggests that ZFD renders *S. mutans* more sensitive to oxidative stress (Aqawi et al., 2021). This suggests that combining ZFD with hydrogen peroxide (H_2_O_2_) may have a synergistic effect on *S. mutans* biofilm control. In addition, ZFD may be used in plaque microecology regulation, as some bacteria such as *Streptococcus sanguinis* could compete with *S. mutans* for H_2_O_2_ (Zhang et al., 2022a). Nevertheless, these hypotheses require further validation.

Antimicrobial resistance is a global concern that threatens public health (Jee et al., 2018). For example, CHX, the most commonly used antimicrobial agent for oral biofilm control, exhibits remarkable antibacterial properties (Van Der Weijden et al., 2015). However, CHX was found to induce a four-fold increase in MIC against *S. mutans* after repeated exposure (Zhang et al., 2021). Our results are consistent with those of previous reports in which *S. mutans* displayed depressed susceptibility to CHX, indicating a potential for CHX resistance. In contrast, our serial passaging experiments demonstrated that prolonged exposure to ZFD did not result in a significant increase in MIC values, indicating a low risk of resistance induction, further highlighting the advantages of ZFD as a promising therapeutic alternative. In addition, according to our experimental experience, CHX can cause a white turbidity when added to the culture medium. This may explain why the CHX group showed higher OD values in the crystal violet and MTT assays but lower viable bacterial cell counts in CFU counting compared with the 0.250 and 0.125 μg/mL ZFD groups.

The primary antimicrobial mechanism of CHX is disruption of the bacterial cytoplasmic membrane, which increases membrane permeability and causes leakage of intracellular contents, ultimately leading to cell death (Van Der Weijden et al., 2015). Consistent with this membrane-damaging mode of action, our SEM images revealed numerous swollen and ruptured cells, as reported in previous studies (Sun et al., 2021). In contrast, as a novel spiropyrimidinetrione-class antibiotic, ZFD targets the GyrB subunit of bacterial DNA gyrase, thereby inhibiting the function of type II topoisomerases, ultimately blocking DNA replication and repair, and leading to bacterial cell death (Oyardi et al., 2024). In our experiments, a fraction of *S. mutans* cells displayed marked surface collapse and shrinkage, while bacterial survival rate did not show any significant change within biofilms under 0.250 μg/mL and 0.125 μg/mL ZFD. Besides, a dramatic growth inhibition of *S. mutans* was observed at these concentrations. Based on the above facts, we hypothesize that *S. mutans* biofilm formation was inhibited, at least partly, through growth inhibition. Whether the DNA gyrase of *S. mutans* was involved in biofilm formation suppression requires further investigation.

In terms of biosafety, *in vitro* cytotoxicity testing using L929 fibroblasts demonstrated that ZFD exhibited no significant cytotoxicity at the concentrations used in this study. Previous reports have indicated that ZFD exhibits favorable tolerability and safety in single-dose therapeutic applications (Taylor et al., 2018). Notably, a previous study reported that ZFD had a lower MIC against *Staphylococcus aureus* at pH 5.5 (a critical pH for enamel demineralization) than at pH 7.0 (Giacobbe et al., 2017). It appears that an acidic environment is more conducive to its antibacterial effect by maintaining its protonation, which is favorable for caries control (Giacobbe et al., 2017). However, this hypothesis warrants further investigation.

Combination therapy is a promising strategy for dental caries control basing on its multiple advantages including reduced drug dosage, lower toxicity, and relieved drug resistance (Gao et al., 2023). One good example is the combination of arginine with NaF, which has shown synergistic effects on biofilm control for caries control (Zheng et al., 2015). CHX is widely regarded as the gold standard antibacterial agent, while NaF is widely used for dental caries prevention because of its well-established roles in remineralization and antibacterial activity (Brookes et al., 2020; Zhang et al., 2022a). Thus, we chose to combine ZFD with CHX or NaF to determine potential synergistic or antagonistic interactions of these combinations on bacterial growth and biofilm formation. Unfortunately, combinations of ZFD with NaF or CHX did not show synergistic effects but showed indifferent effects. However, these indifferent effects still indicate ZFD’s compatibility with CHX or NaF as a potential adjunctive agent (Martins et al., 2025).

Our study has certain limitations. Dental plaque biofilms are more complex, and the oral environment in which they exist is extremely complicated (Campus et al., 2025). Although *S. mutans* biofilms have been commonly used as dental caries-associated *in vitro* research models due to their markedly strong cariogenicity, more complex biofilm models, such as saliva- or dental plaque-originated biofilms, should be employed. Besides, the establishment of rodent models of dental caries would help assess ZFD’s stability, safety, and anticaries efficacy. Moreover, based on its suppressive effects on *S. mutans* biofilm formation, ZFD could potentially be loaded into diverse nanoplatforms that can attach to the tooth surface for caries prevention by drug release. Additionally, ZFD could be employed to endow dental filling materials with antibacterial properties for secondary caries prevention.

## Conclusion

Since ZFD can suppress *S. mutans* biofilm formation and its cariogenic virulence, has low cytotoxicity, and has a minimal risk of resistance induction, it possesses considerable potential for application in caries prevention through biofilm control.

## Data Availability

The raw data supporting the conclusions of this article will be made available by the authors, without undue reservation. Further inquiries can be directed to the corresponding authors.
